# Complete Genome Sequence of West Nile Virus Isolated from Alappuzha District, Kerala, India

**DOI:** 10.1128/genomeA.00230-13

**Published:** 2013-05-16

**Authors:** Anukumar Balakrishnan, Datta K. Butte, Santhosh M. Jadhav

**Affiliations:** National Institute of Virology, Kerala Unit, Government TD Medical College Hospital Complex, Alappuzha, Kerala, Indiaa;; National Institute of Virology, Pune, Maharashtra, Indiab

## Abstract

West Nile virus belongs to the *Flaviviridae* family, transmitted by vector mosquitoes. Here, we reported the complete genome sequence of West Nile virus isolated from human samples during an acute encephalitis outbreak in Kerala, India. Phylogenetic analysis revealed that the virus genome clusters into genetic lineage 1, clade 1a.

## GENOME ANNOUNCEMENT

West Nile virus (WNV) is an arthropod-borne virus transmitted by vector mosquitoes. The virus belongs to the genus *Flavivirus* Japanese encephalitis antigenic complex, in the *Flaviviridae* family. The seroprevalence of WNV antibodies in a population indicated the presence of this virus in India (1, 2). Later, WNV isolates from mosquito, bat, and human specimens confirmed the circulation of this virus in India (3). Phylogenetic analysis of the partial genome indicated that Indian isolates of WNV belong to genetic lineage 1 (clade 1c) (4). Subsequently, the Indian isolates were classified into a newer genetic group, lineage 5 (5). In humans, WNV generally produces mild fever, and it sometimes can produce encephalitis, meningitis, and other infections in adult patients.

We reported an outbreak of acute encephalitis syndrome (AES) from May through July 2011 in Kerala, India. Per our records, 208 AES cases, with 4 deaths, were recorded. Among 208 AES cases, 69 (33%) were in patients belonging to the pediatric age group (those aged <15 years) and 139 (66.82%) were in adults (aged >15 years). Clinically, the patients had high fever with headache and one or more of the following symptoms: neurological deficit, altered sensorium, disorientation, irritability, neck rigidity, and vomiting. No clustering of the cases was noticed in the affected area. A virus neutralization assay confirmed that the etiology of the outbreak was WNV. The virus was isolated from a serum sample (sample identification no. 1048813) from a patient with acute fever. The patient serum was diluted (1:10) in minimum essential medium (MEM) consisting of 1% fetal calf serum (FCS) and gentamicin (50 µg/ml), and it was inoculated into the Vero E6 cell line. The cell line was observed for 7 days for any cytopathic effects (CPE). WNV was able to be isolated after a second blind passage in Vero E6 cells.

Total RNA was extracted from the culture supernatant using a QIAamp viral RNA minikit (Qiagen, Germany). Previously described primers were used for the amplification of the full genome (6). cDNA was synthesized using gene-specific primers and avian myeloblastosis virus (AMV) reverse transcriptase. Fifteen fragments of the full genome (size, ~0.5 to 1.3 kb) were amplified from synthesized cDNA, and the PCR product was purified and sequenced. The genome of WNV is 10,948 bp long.

The raw sequence data were assembled using MEGA software (version 5.0). Sequence alignments and phylogenetic trees were generated using the MEGA program (7). The complete genome of the Kerala isolate comprises 10,948 nucleotides, with an overall G+C content of 50.92%. A polyprotein is encoded by 10,293 nucleotides with a single open reading frame (ORF). The ORF region was subjected to a BLAST search with available WNV nucleotide sequences from GenBank. The BLAST result showed that the Kerala isolate has the highest (98.62%) identity to the Russian isolate Ast01-182 (GenBank accession no. DQ411030). We aligned the Kerala WNV isolate whole-genome sequences with available WNV sequences from GenBank. The neighbor-joining method was employed to construct the phylogenetic tree. The tree indicated that the Kerala isolate belongs to lineage 1 (clade 1a). This report will help in understanding the molecular characteristics and epidemiology of WNV in southern India.

### Nucleotide sequence accession number.

The complete genome sequence of the WNV Kerala strain has been deposited at GenBank under the accession no. KC601756.
